# Monitoring of patients treated with particle therapy using positron-emission-tomography (PET): the MIRANDA study

**DOI:** 10.1186/1471-2407-12-133

**Published:** 2012-04-03

**Authors:** Stephanie E Combs, Julia Bauer, Daniel Unholtz, Christopher Kurz, Thomas Welzel, Daniel Habermehl, Thomas Haberer, Jürgen Debus, Katia Parodi

**Affiliations:** 1 Department of Radiation Oncology, University Hospital of Heidelberg, INF 400, 69120 Heidelberg, Germany; 2 Heidelberger Ionenstrahl Therapiezentrum (HIT), INF 450, 69120 Heidelberg, Germany

## Abstract

**Background:**

The purpose of this clinical study is to investigate the clinical feasibility and effectiveness of offline Positron-Emission-Tomography (PET) quality assurance for promoting the accuracy of proton and carbon ion beam therapy.

**Methods/Design:**

A total of 240 patients will be recruited, evenly sampled among different analysis groups including tumors of the brain, skull base, head and neck region, upper gastrointestinal tract including the liver, lower gastrointestinal tract, prostate and pelvic region. From the comparison of the measured activity with the planned dose and its corresponding simulated activity distribution, conclusions on the delivered treatment will be inferred and, in case of significant deviations, correction strategies will be elaborated.

**Discussion:**

The investigated patients are expected to benefit from this study, since in case of detected deviations between planned and actual treatment delivery a proper intervention (e.g., correction) could be performed in a subsequent irradiation fraction. In this way, an overall better treatment could be achieved than without any in-vivo verification. Moreover, site-specific patient-population information on the precision of the ion range at HIT might enable improvement of the CT-range calibration curve as well as safe reduction of the treatment margins to promote enhanced treatment plan conformality and dose escalation for full clinical exploitation of the promises of ion beam therapy.

**Trial Registration:**

NCT01528670

## Background

In comparison to conventional external beam radiotherapy with photon and electron radiation, ion therapy may offer superior conformation of the dose delivery to the tumour, with improved sparing of the surrounding healthy tissue and critical organs. This is mainly due to the favourable energy deposition, which can be concentrated in a few millimeters narrow region (the Bragg-peak) at an adjustable depth [1]. However, exploitation of this selectivity to its full extent in the clinical practice is still hampered by uncertainties in the knowledge of the beam range in the patient, resulting in the usage of generous safety margins as well as avoidance of beam portals stopping directly in front of critical organs. In fact, the complete stopping of the primary ion beam in the patient prevents the applicability of conventional quality assurance techniques like electronic portal imaging [2], which are well established in clinical radiotherapy practice with photon radiation. Hence, treatment planning algorithms can only be based on models and data which are accurately validated in tissue-equivalent phantoms [3,4], but no direct in-vivo verification within the patient is currently feasible.

Nowadays, Positron-Emission-Tomography (PET) offers the only technically feasible method for an indirect three-dimensional (3D), in-vivo, non-invasive verification of ion treatment during or shortly after irradiation [5-11]. The method is based on the detection of the transient β^+^-activity which is formed as a by-product of the therapeutic irradiation (i.e., without administering any radio-tracer to the patient) in nuclear reactions between the incoming ions and the target nuclei of the irradiated tissue. By correlating the measured activity with the planned treatment and the actual patient anatomy as given by a computed tomogram (CT), it is possible to infer valuable information on the actual dose delivery. As activity and dose are different physical quantities, an additional comparison of the acquired PET images with a corresponding activation pattern deduced from the planned treatment and the specific time course of irradiation and imaging is strongly recommended [6,11]. This expectation can be obtained from a detailed Monte Carlo calculation of the ion beam transport and interaction within the patient [6,12-14], combined with the estimation of the fraction of activity carried away from the place of production by physiological processes such as perfusion, generally referred to as "biological washout" [6].

The technical implementation of PET monitoring can be performed using dedicated (typically limited angle) instrumentation directly located at the treatment site for in-beam [11] or in-room [8] acquisition, or using commercial nuclear medicine PET and PET/CT scanners installed closeby to the treatment unit for offline imaging [6,7,9,10]. In all cases, up-to-date anatomical information of the patient can be obtained from control CTs (cone-beam or full-ring scanner) which are acquired in regular intervals as a standard of care to evaluate correct patient positioning or to detect any anatomical changes during the course of the fractionated treatment in particle therapy. Therefore, no additional CT-imaging is required for correlation with PET measurements; however, when an additional CT is available, anatomical changes may be monitored and detected.

Promising experience in PET imaging of proton and carbon ion therapy has been so far obtained for more than 50 patients monitored after passive proton treatments in USA and Japan, as well as more than 400 patients monitored during scanned carbon ion beam irradiation at GSI Darmstadt, Germany [5-11]. In particular, the GSI pilot project demonstrated the value of PET for improving the accuracy of the semi-empirical CT-range calibration curve employed by the treatment planning system as well as for detecting and quantifying deviations between planned and actual treatment delivery due to patient misalignments or anatomical changes over the course of fractionated therapy [5,11,15]. Therefore, this clinical experience clearly indicates the potential value of the method especially in the starting phase of a new facility such as the Heidelberger Ionenstrahl Therapiezentrum (HIT) [16]. For this purpose, a commercial PET/CT scanner has been installed at HIT in a dedicated room closeby to the treatment area. However, the results from different therapy facilities cannot be directly translated to the HIT center due to the differences in the clinical protocol (fraction dose, number of portals), ion species (p,^12^C), beam-delivery system (passive scattering or active pencil-beam scanning), detector properties (full-ring or limited angle PET scanner, detection technology), imaging protocol (in-beam, in-room or off-beam data acquisition [17]), as well as the limited number of the so far investigated tumor types and sites. Therefore, a pilot clinical study is needed at HIT in order to identify the patient population which may benefit from PET imaging, as well as extract population-based information on the reliability of the delivered beam range for the available ion species in different tumour locations.

In the proposed clinical study MIRANDA (**M**on**i**toring of Patients t**r**eated with P**a**rticle Therapy using Positro**n**-Emission-Tomography) patient selection will be particularly addressed to those more crucial situations where utmost precision in the delivery of the planned dose with optimal sparing of the surrounding critical structures is mandatory. Especially in those cases where the ions have to penetrate highly inhomogeneous tissue, the precision of the intended dose deposition can be severely undermined by uncertainties of the treatment planning algorithms and minor positioning errors. Thus, the proposed PET method might offer a useful tool for assessing unpredictable deviations between planned and actual treatment. Moreover, PET-based verification might be beneficial for patients having metallic implants, where known shortcomings of the treatment planning algorithms in combination with artifacts of the CT-based patient model are of major dosimetric concern in ion beam therapy [18].

The participating patients are expected to benefit from this study, as in the eventuality of detected discrepancies a timely intervention could be undertaken, thus guaranteeing an improved treatment quality than without PET monitoring. Moreover, site-specific patient-population information on the ion range precision at HIT might enable in-vivo validation of the CT-range calibration curve as well as safe reduction of the treatment margins to promote enhanced treatment plan conformality for full clinical exploitation of the ballistic selectivity offered by ion beam therapy.

## Methods and Design

### Aims of the Study

The purpose of this clinical study is to investigate the clinical feasibility and effective benefit of off-line PET for quality assurance of scanned proton and carbon ion beam therapy for a large patient population.

The specific aims are to

1) Acquire PET/CT scan data on patients undergoing external beam ion therapy for a representative population of different tumour sites in order to be able to conclude which patients might in future benefit from PET/CT monitoring.

2) Analyze these data to determine the accuracy with which the beam range can be monitored and the actually delivered dose can be estimated.

3) Analyze these data (acquired during at least three fractions at the beginning, the middle and the end of the regular treatment course) to determine whether quantitative changes of the PET signal after delivery of the same treatment fraction dose can be indicators of physiological modifications connected to tumour response.

4) Assess the possibility of using PET/CT monitoring for quality assurance and adaptive strategies in scanned ion beam therapy.

### Procedure to be evaluated

The PET/CT scanner to be used is a CE-labelled medical product (Siemens Biograph mCT 40). The procedure will include acquisition of a control CT for anatomical reference as well as list-mode detection of the β^+^-activity resulting from the standard therapeutic irradiation. The CT scan is performed for patient position verification as well as monitoring of anatomical changes during the course of radiotherapy and is considered standard of care. Therefore, no trial specific CT imaging will be acquired and patients will not be exposed to additional experimental doses of ionizing radiation. The PET signal to be measured is a by-product of the therapeutic ion beam irradiation, hence no additional radioactive tracer is administered to the patient. The treatment position will be reproduced at the PET/CT scanner using the same immobilization equipment used for the therapeutic irradiation, which is compatible with standard diagnostic CT and PET imaging.

The activity levels to be expected in the study are orders of magnitude lower than in the typical operation condition of the commercial PET device, which is designed for application to nuclear medicine tracer imaging. Therefore, the PET data acquisition will be performed in one single bed position and last over 30 minutes to collect sufficient counting statistics for imaging, following the same clinical protocol reported by other investigators in previous clinical trials in USA [6,10]. After the first clinical data will be collected, the available iterative reconstruction schemes of the PET scanner will be applied and the resulting images will be compared in order to identify the optimal image reconstruction settings for optimal imaging performances at the expected low statistics levels. The so identified optimal image reconstruction settings will be applied to process all the clinical data acquired in this study.

The reconstructed PET images co-registered to the control CT (using the CE-labelled software provided by the manufacturer) will be finally analysed in comparison to the planned treatment to infer information on the correctness of the actually delivered therapeutic irradiation. An additional comparison with an expected β^+^-activity distribution will be pursued [19].

### Therapeutic and Diagnostic Modes of Action

By taking part in the MIRANDA study patients are offered the possibility to determine the applied dose more precisely, since by using the PET/CT for monitoring the activation produced by the particle beam can be visualized. The potential benefit for the future of radiotherapy is to implement PET/CT in clinical routine for dose monitoring possibly providing a useful tool for necessary corrections in treatment planning or patient positioning (adaptive particle therapy).

### Side effects and risks as well as potential additional exposures during study treatment

The β^+^-activity is induced by the therapeutic irradiation; therefore, no additional exposure of the patient is required for the PET measurements. To carry out the investigation with accurate anatomical co-registration, this study is performed using a PET/CT. The additional control CT enables the researchers to explore also anatomical modifications compared to the initial imaging data acquired for treatment planning. These control CTs are performed on a regular basis during the course of precision radiotherapy for verification of patient positioning and to monitor any anatomical changes to the tumor and to normal tissue. Therefore, these CT scans can be considered standard of care, and patients will not be exposed to any additional ionizing radiation.

This study will cause the prolongation of the selected treatment sessions, because of the time needed for the transport of the patient to the remote PET/CT scanner (about 5 min) and for the PET/CT acquisition (about 30 min). The latter is mainly determined by the required 30 minutes of PET acquisition, being the CT imaging typically completed in less than 1 min.

### Study Design

Open, mono-centric, non-randomized, prospective study to evaluate PET-CT monitoring after particle therapy for position verification, dose deposition monitoring and for the implementation of adaptive strategies for particle therapy

### Inclusion Criteria

• The patient is treated at the Heidelberger Ionenstrahl Therapiezentrum (HIT) with protons or carbon ions.

• During the radiotherapeutic treatment patient positioning and anatomy is verified using validated radiological imaging such as cone beam CT, X-ray or conventional CT (Reference-Imaging as described above).

• The patient is at least 18 years of age and is able to give informed consent.

• The patient has been informed about the aims and the content of the study.

Within the study protocol, no additional radiological imaging techniques will be performed. Therefore, the patient will not be exposed to any additional irradiation.

### Exclusion Criteria

• No informed consent to take part in the study.

• Medical reasons that impair the patients from being in the supine position for the data acquisition time, e.g. pain.

• Non-compliance of the patient.

### Study plan

A patient fulfilling the inclusion criteria will be informed about the background of this study and will be offered participation. Thereafter, an information brochure about the study will be handed out to the patient, and the patients will be instructed about the study plan and procedures. Thereafter, the patient can give informed consent. This informed consent will be documented in a written form (signature on informed consent form).

After the patient has given informed consent, patients are included into the study. The study will include procedures as follows:

a. Right after a regular treatment fraction the patient will be transferred to the PET/CT scanner located on the same floor within few meters distance from the treatment sites in the HIT building.

b. A PET/CT scan will be taken. For this examination the patient will remain fixated in the immobilization device used during treatment delivery. The average acquisition time will be in the order of 30 minutes.

The procedure will be repeated for a total of at least three treatment fractions, one being selected in the first week of treatment, one in the middle of the treatment period and one during the last week of therapy.

The study will cause prolongation of the selected treatment sessions due to the transport to the remote PET/CT scanner (about 5 min) and the PET/CT acquisition (about 30 min). All subsequent data analysis will be performed without interaction with the patient.

The data will be pseudonymized and analysed.

Concomitant treatment

No concomitant treatment is part of the study protocol.

### Criteria for withdrawal

#### Individual criteria

• Pain in supine position

• Agitated or restless patient

• Discontinuation of study participation by the patient

#### General withdrawal criteria for the study

There are no general withdrawal criteria for the study protocol

### Statistical Design

Feasibility and value of post-radiation PET imaging for verification of therapeutic ion beam delivery should be validated for different organ regions. Therefore, different analysis groups will be evaluated including tumors of the brain, skull base, head and neck region, upper gastrointestinal tract (upper GI), lower gastrointestinal tract (lower GI), prostate and pelvic region. For optimal calculations and verification, 30 patients per anatomic region will be analyzed. This amounts to a total patient number of 240 patients.

After acquisition of the data, correlation of the measured activity with the acquired CT imaging for patient position and anatomy will be performed. Additionally, correlation with the calculated dose plan within the Siemens Treatment Planning System (TPS) will be performed. Potential correction vectors consisting of positional changes will be determined based on the newly acquired activity images and the imaging that had been done for treatment planning.

Correlation of the measured activity level with the applied dose and pattern of RBE-values (relative biological effectiveness) determined during the biological treatment planning for particle therapy will be performed.

As activity and dose are known to be different physical quantities, an additional calculation of the expected β^+^-activity distribution will be pursued, provided that the computational tool used for this purpose is verified to deliver a corresponding calculation of dose distribution in agreement with the certified clinical TPS.

To assess whether the calculated dose and activity distribution are different from the measured activity corresponding to the applied dose distribution, a *t*-test as a one-sample location test can be used („is the difference different from zero").

### Ethical and legal aspects

The procedures set out in this trial protocol, pertaining to the conduct, evaluation, and documentation of this trial, are designed to ensure that all persons involved in the trial follow the guidelines of Good Clinical Practice (GCP) and the ethical principles described in the applicable version of the Declaration of Helsinki (2008 Version of the Declaration of Helsinki, adopted at the 59th WMA General Assembly, Seoul, October 2008), as well as in accordance with the „Berufsordnung für Ärztinnen und Ärzte der Landesärztekammer Baden-Württemberg"in the most recent version.

The trial will be carried out in adhering to local legal and regulatory requirements.

The study plan was submitted to the Institutional Review Board (IRB)/independent Ethics Committee (EC) of the Medical Faculty Heidelberg, and official approval was obtained.

Participation of a patient in this study is voluntary. A subject may voluntarily discontinue participation in this study at any time at their own request. Before study entry, patients will be informed by the written information brochure as well as in orally about the planned procedures within this study, especially about potential benefit for their health or potential risks. Informed consent will be documented by the patient's signature on the informed consent form.

All patients will be informed prior to initiation of study treatment concerning the aims of the study, especially with respect to any risks or side effects of the treatment, as well as the potential use of study participation for the overall treatment course. Informed consent will be documented as written informed consent by signature on the informed consent sheet. If the subject withdraws from the trial and also withdraws consent for disclosure of future information, no further evaluations should be performed, and no additional data should be collected. In all cases, the reason for withdrawal must be recorded. In case of withdrawal of a subject at his/her own request, the reason should be asked for as extensively as possible and documented. All data acquired within this study will be deleted, except if the patients allows for further evaluation of the data and inclusion into the final analysis.

The data obtained in the course of the trial will be treated pursuant to the Federal Data Protection Law (Bundesdatenschutz- bzw. Landesdatenschutzgesetz, BDSG, LDSG).

Trial findings stored on a computer will be stored in accordance with local data protection law and will be handled in strictest confidence. For protection of these data, organizational procedures are implemented to prevent distribution of data to unauthorized persons. The appropriate regulations of local data legislation will be fulfilled in its entirety.

No third party will have access to the patient data.

## Discussion

**Figure 1:**
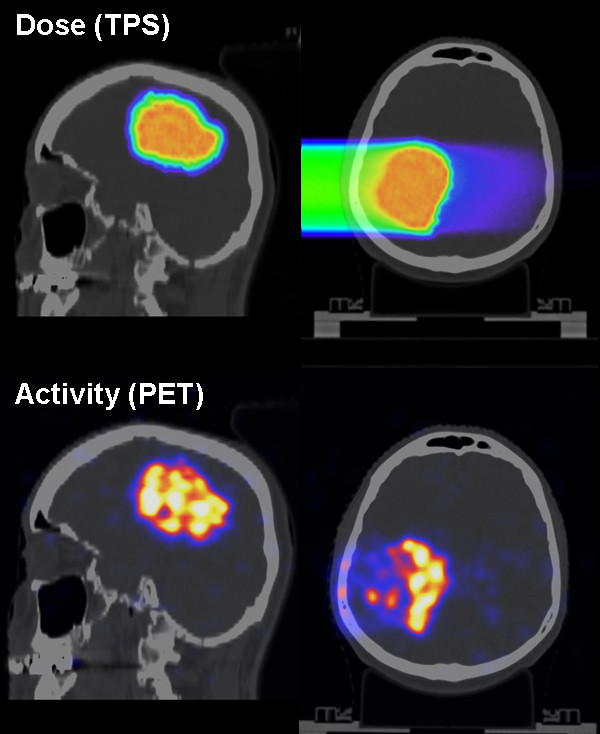
**Patient undergoing a PET/CT measurement after scanned ion irradiation at HIT**. The patient was treated for a primary brain tumour with a carbon ion boost. The top panel depicts the dose planned by the commercial TPS overlaid onto the planning CT in axial and sagittal views. The bottom panel depicts the PET image acquired ca. 8 minutes after irradiation and overlaid onto the control CT data, merged and visualised approximately in the same views as the top raw.

An example of the first post-radiation PET/CT imaging of a patient undergoing a scanned carbon ion boost treatment of a glioblastoma tumour at HIT is illustrated in Figure 1[20]. After completion of a therapeutic treatment fraction (3 GyE), the patient was walked to the PET/CT scanner and immobilised using the same equipment as for therapy. The 30 min PET acquisition could be started about 8 min after single-field irradiation. The depicted data refer to the prescribed dose from the planning system (top) and the merged PET/CT images (bottom). Despite the noise of the low-statistics PET images, a promising correlation between the detected activation and the planned dose is observed. In particular, this first clinical case strongly supports the possibility to visualize the delivered treatment and to confirm in-vivo the planned ion range. Nevertheless, a more detailed quantitative analysis as well as the additional comparison with the expected pattern of induced activity is underway and will be separately reported [19,21].

From the encouraging initial results, it can be expected that the proposed MIRANDA clinical study will enable quantitative assessment of the feasibility and value of offline PET-based in-vivo treatment verification for promoting high precision ion beam therapy at HIT for different treatment sites and ion species.

## Abbreviations

CT: Computer Tomography; PET: Positron-Emission-Tomography; EC: Ethics Committee; GCP: Good Clinical Practice; GSI: Gesellschaft für Schwerionenforschung; Gy: Gray; GyE: Cobalt Gray equivalent; HIT: Heidelberger Ionenstrahl Therapiezentrum; LET: linear energy transfer; LLUMC: Loma Linda University Medical Center; MGH: Massachusetts General Hospital.

## Competing interests

The authors declare that they have no competing interests.

## Authors' contributions

KP and SEC have developed the study concept. SEC, KP, TH and JD wrote the study protocol and obtained ethics approval. SEC, DH, TW and JD will provide patient care. KP, SEC, JB, CK, DU and JD will implement the protocol and oversee collection of the data. All authors contributed to and approved the final manuscript.

## Pre-publication history

The pre-publication history for this paper can be accessed here:

http://www.biomedcentral.com/1471-2407/12/133/prepub
